# Design and Characterization of GelMA Nanogels (nanoGelMA) via Desolvation and Photopolymerization for Drug Delivery Applications

**DOI:** 10.3390/pharmaceutics18070812

**Published:** 2026-06-30

**Authors:** Roberta Pappalardo, Rossella Laurano, Claudio Cassino, Stefano Bianchi, Valeria Chiono, Gianluca Ciardelli, Monica Boffito

**Affiliations:** 1 Department of Mechanical and Aerospace Engineering, Politecnico di Torino, 10129 Turin, Italy; rossella.laurano@polito.it (R.L.); valeria.chiono@polito.it (V.C.); gianluca.ciardelli@polito.it (G.C.); monica.boffito@polito.it (M.B.); 2 Department of Life Sciences, Università di Modena e Reggio Emilia, 41125 Modena, Italy; 3 Institute for the Chemical-Physical Processes (IPCF), National Research Council (CNR), 56124 Pisa, Italy; 4 Department of Science and Technological Innovation and Magnetic Resonance Platform (PRISMA-UPO), Università del Piemonte Orientale, 15121 Alessandria, Italy; claudio.cassino@uniupo.it

**Keywords:** gelatin methacryloyl, nanogels, nanoGelMA, drug delivery system

## Abstract

**Background/Objectives:** Micro- and nano-scale hydrogels (microgels and nanogels) have attracted increasing attention as carriers for drug delivery due to their high-water content, responsiveness to external stimuli, tunable properties, and versatility. In this work, gelatin methacryloyl (GelMA) with a medium degree of methacryloylation (DoM ca. 60%) was ad hoc synthesized as a constituent material for nanogel production. For the first time in the literature, GelMA-based nanogels (nanoGelMA) were developed through an optimized two-step desolvation method combined with photo-crosslinking. **Methods:** The influence of key process parameters, including the pH, volume of desolvating agent, photo-initiator concentration, and UV exposure time, was systematically investigated to identify optimal conditions for nanoGelMA preparation. To assess its potential as a drug delivery nanocarrier, the nanoGelMA was loaded with ibuprofen (IBU) as a model anti-inflammatory drug via in situ encapsulation during nanogel preparation. **Results:** The formulated nanoGelMA exhibited an average hydrodynamic diameter (d) of ca. 250 nm, a polydispersity index of 0.2, and a production yield of approximately 30%. The nanogels demonstrated stability in water and in phosphate buffer at pH 5 over 96 h, while exhibiting significant swelling in physiological-like conditions and enzymatic degradation (d of ca. 421 ± 91 nm and 609 ± 182 nm at 96 h, respectively). The cytocompatibility evaluation demonstrated high cell viability (86–96%) of the nanoGelMA at different concentrations (1–5 mg/mL). IBU-loaded nanoGelMA particles were successfully developed via direct drug encapsulation during nanogel formation, achieving a maximum encapsulation efficiency of ca. 30%, and exhibited environment-responsive release, with kinetics modulated by the ionic strength, pH, and enzymatic activity. **Conclusions:** Overall, the nanoGelMA developed herein represents a promising nanogel platform with great potential for the development of advanced and controlled drug delivery therapies.

## 1. Introduction

Nanogels are three-dimensional submicron hydrogels formed by crosslinked polymer chains, which retain the highly hydrated nature and liquid absorption capacity of hydrogels while exhibiting the nanoscale size of nanoparticles. Due to these features, they have been widely investigated in the biomedical field, particularly as drug delivery systems (DDSs) and for theranostic applications [1,2]. In this context, nanogels are considered innovative and biocompatible nanocarriers, offering high stability, biodegradability, low toxicity, and reduced immunogenicity, thereby overcoming several limitations of conventional therapies. As nanocarriers, the main advantage of nanogels lies in their high loading capacity for bioactive molecules, enabled by the presence of functional groups along the constituent polymer chains, which promote interactions (electrostatic forces, hydrogen bonding, and Van der Waals interactions) with several drugs and biomolecules. Moreover, their strong affinity for aqueous environments allows them to absorb fluids and swell, facilitating the encapsulation and controlled release of a wide range of therapeutic agents, including proteins, peptides, biomolecules of different molecular weights, and poorly water-soluble drugs. In addition, their small size, high surface area, and stimuli-responsive behavior enable tissue penetration, targeted delivery, and sustained drug release. All these features make nanogels particularly suitable for applications in gene therapy, chemotherapy, diagnosis, and regenerative medicine [3,4,5]. Among the different materials used for nanogel formulation, natural proteins, such as elastin, collagen, gelatin, and silk fibroin, show great promise for nano-system preparation due to their intrinsic biocompatibility and biodegradability. Furthermore, they also bear functional groups (e.g., amino and carboxylic acid groups, hydroxyl groups, and thiols) that confer responsiveness to environmental stimuli or allow further functionalization, enabling active targeting strategies [6,7]. In particular, gelatin, a hydrolyzed derivative of collagen, is widely used as a biomaterial due to its low toxicity, high availability, and cost-effectiveness and has been commonly investigated for the development of both macroscopic hydrogels and nanocarriers. So far, different approaches have been reported in the literature for the preparation of gelatin-based nanoparticles, including desolvation, emulsification, and nanoprecipitation, although each method presents specific limitations [8,9]. The desolvation technique relies on the self-assembly of gelatin chains in the presence of a desolvating agent (e.g., acetone or alcohol), leading to nanoparticle formation (200–300 nm). However, the subsequent crosslinking step, typically performed using glutaraldehyde, may reduce biocompatibility [10,11,12]. Using an emulsification method, gelatin nanoparticles (100–200 nm) are prepared via the dispersion of an aqueous gelatin phase into an oil phase followed by stabilization with glutaraldehyde or genipin. The use of organic solvents and the need for additional purification steps have been reported as the main limitations [13,14,15]. Nanoprecipitation represents a simpler alternative to produce gelatin nanoparticles (200–350 nm) as result of the dropwise addition of a gelatin solution into a non-solvent, although it generally requires a high amount of stabilizers [16,17]. In recent years, gelatin-based nanoparticles and nanogels have been widely investigated for drug-delivery applications, particularly in cancer therapy [18,19,20,21]. For instance, Fatima et al. developed injectable doxorubicin (DOX)-loaded gelatin nanoparticles using a two-step desolvation method and glutaraldehyde as a crosslinker, achieving an encapsulation efficiency of approximately 55–65%. The system exhibited pH-responsive behavior, with enhanced drug release under acidic conditions typical of tumor environments [22]. Similarly, Vaghasiya et al. designed cisplatin-loaded gelatin nanoparticles for lung cancer treatment, achieving an encapsulation efficiency of ca. 74% and enabling targeted delivery through surface functionalization with concanavalin A, a glycoprotein able to selectively bind to mannose receptors overexpressed in tumor tissues. The release of cisplatin was shown to be enzyme-responsive, being triggered by matrix metalloproteinases secreted in the tumor microenvironment [23]. A comparable approach was reported by Zhou et al., who developed paclitaxel (PTX)-loaded gelatin nanoparticles via nanoprecipitation, followed by functionalization with bovine serum albumin (BSA) to achieve active targeting. The system demonstrated enzyme-triggered drug release and significant antitumor activity both in vitro and in vivo [24]. In addition to anticancer applications, gelatin-based nanocarriers have also been investigated for antibacterial and antifungal therapies, as demonstrated by the development of antibiotic-loaded nanoparticles for improving the delivery of poorly cell-penetrating drugs such as spectinomycin and chloramphenicol [25]. Furthermore, their potential for gene therapy has been highlighted by Andrée et al., who reported the use of gelatin nanoparticles as non-viral vectors for mRNA delivery, showing the role of the nanoparticle surface charge in cellular uptake and payload binding [26]. Despite these promising results, conventional gelatin-based nanocarriers for drug-delivery applications present some limitations related to the use of potentially toxic crosslinking agents for their preparation and limited control over network structure. To address these challenges, in this work, a customized gelatin methacryloyl (GelMA) was synthesized and used as constituent material for designing GelMA-based nanogels (nanoGelMA). A detailed comparison among the nanoGelMA formulated in this study and existing gelatin- and gelatin methacryloyl-based nanoparticle systems is provided in Table S1. To the best of our knowledge, this work reports, for the first time, the preparation of a nanoGelMA using an optimized methodology, combining a two-step desolvation technique and photopolymerization. The influence of key process parameters, including the pH, volume of desolvating agent, photo-initiator concentration, and light exposure time, was investigated. The resulting nanoGelMA particles were characterized in terms of size, Zeta potential, production yield, stability in different aqueous environments, and cytocompatibility according to regulations. Furthermore, the potential application of nanoGelMA as a drug delivery system was evaluated through the encapsulation and release of ibuprofen as a model anti-inflammatory molecule.

## 2. Materials and Methods

### 2.1. Synthesis of Gelatin Methacryloyl (GelMA)

Custom-made gelatin methacryloyl (GelMA) was synthesized according to a protocol already reported in the literature with some modifications [27]. Briefly, 10 g of gelatin type A from porcine skin (~300 g Bloom, pI 7.0–9.0, product code G1890, Sigma Aldrich, Milan, Italy) was solubilized in warm phosphate-buffered saline solution (PBS, pH 7.4) at a 10% *w*/*v* concentration and stirred at 60 °C until complete solubilization. Then, to synthesize GelMA with a medium degree of methacryloylation, 0.16 mL of methacrylic anhydride (MA, Sigma Aldrich, Milan, Italy) per gram of gelatin was added dropwise, and the reaction mixture was stirred at 50 °C for 3 h. Subsequently, PBS was added to terminate the reaction (at a 5:1 *v*/*v* ratio with respect to the initial PBS volume used to dissolve gelatin). Finally, the resulting solution containing GelMA was transferred into dialysis membrane tubes (MWCO 12–14 kDa, Carlo Erba Reagents S.r.l., Cornaredo, Italy) and dialyzed against demineralized water (Milli-Q Direct Q^®^ 8 UV system, Merck Millipore, Milan, Italy) for one week (the dialysis medium was refreshed three times/day). Lastly, the GelMA solution was freeze-dried (ALPHA 2–4 LSC, Martin Christ Gefriertrocknungsanlagen GmbH, Osterode am Harz, Germany) and stored under vacuum at 4 °C until use.

### 2.2. Chemical Characterization of GelMA

#### 2.2.1. Attenuated Total Reflectance–Fourier Transform Infrared (ATR-FTIR) and Proton Nuclear Magnetic Resonance (^1^H NMR) Spectroscopies

ATR-FTIR spectroscopy was performed using a Perkin Elmer Spectrum 100 equipped with an ATR accessory (UATR KRSS, PerkinElmer Inc., Shelton, CT, USA) with a diamond crystal. GelMA was analyzed at room temperature in the spectral range 4000–600 cm^−1^ (resolution 4 cm^−1^, 32 scans). As a reference, the gelatin spectrum was also recorded according to the same protocol. The spectra were analyzed and compared using the Perkin Elmer Spectrum 10 Software (PerkinElmer Inc., Waltham, MA, USA).

^1^H NMR spectroscopy was performed using a Bruker Avance NEO spectrometer (Bruker Biospin AG, Fällanden, Switzerland) equipped with a 11.75 T superconducting magnet (500 MHz ^1^H Larmor frequency) and a Bruker multinuclear SMARTProbe (Bruker Biospin AG, Fällanden, Switzerland). Samples were prepared by solubilizing 10 mg of gelatin or GelMA in 0.75 mL of deuterium oxide (D_2_O, 99.8%, Sigma Aldrich, Milan, Italy). The ^1^H NMR spectra were obtained as an average of 24 scans (3.5 s relaxation time). Spectra were elaborated using MNova 6.0 software (Mestrelab Research S.L., Santiago de Compostela, Spain).

#### 2.2.2. Ninhydrin Assay

The degree of methacryloylation (DoM) of the synthesized GelMA was indirectly evaluated through the Ninhydrin assay (commercially available as the Kaiser test kit, Sigma Aldrich, Milan, Italy). In detail, 2.5 mg of gelatin or GelMA was dissolved in 240 μL of double-distilled water (ddH_2_O) and, after complete solubilization, the Kaiser test reagents were added according to the supplier’s instructions (75 μL of phenol solution, 80% in ethanol, 100 μL of KCN in H_2_O/pyridine, and 75 μL of Ninhydrin, 6% in ethanol). Samples were incubated at 120 °C for 5 min and afterwards were diluted by adding 360 μL of ethanol. Then, sample absorbance was measured with a UV-visible spectrophotometer (Perkin Elmer, Lambda 365, PerkinElmer Inc., Waltham, MA, USA) in the spectral range 400–700 nm. The DoM was calculated according to Equation (1):

(1) $$DoM\left(\%\right)=\frac{A_{gelatin}-A_{GelMA}}{A_{gelatin}}\times 100$$

where *A_gelatin_* and *A_GelMA_* indicate the gelatin and GelMA absorbance measured at 570 nm, respectively. Tests were conducted in triplicate, and results were reported as the average value ± standard deviation.

### 2.3. Formulation of Nano-Systems Based on GelMA (nanoGelMA)

Photo-cured gelatin methacryloyl-based nano-systems (nanoGelMA) were formulated through a two-step desolvation method that was optimized starting from the protocol for gelatin nanoparticles preparation already reported by Coester et al [28]. In detail, 250 mg of GelMA was dissolved in ddH_2_O at a 5% *w*/*v* concentration and stirred at 45 °C. After complete solubilization, acetone at a 0.5:1 *v*/*v*, 0.75:1 *v*/*v*, 1:1 *v*/*v*, or 1.5:1 *v*/*v* ratio with respect to ddH_2_O was rapidly added to induce the desolvation and sedimentation of high molecular weight GelMA (HMW GelMA). After 20 min, the supernatant was discarded and 5 mL of ddH_2_O was added to solubilize the HMW GelMA. Then, the pH of the resulting solution was adjusted to 12 with 0.1 M NaOH or to 4.5, 3.5 and 2.5 using 0.1 M HCl, at 45 °C. Subsequently, HMW GelMA was desolvated again via the dropwise addition of acetone at 2:1 *v*/*v*, 3:1 *v*/*v*, or 4:1 *v*/*v* ratio with respect to ddH_2_O, under vigorous stirring (600 rpm) at 45 °C. Lastly, 1 mL of a lithium phenyl-2, 4, 6-trimethylbenzoylphosphinate (LAP, TCI Chemicals Zwijndrecht, TCI EUROPE N.V., Zwijndrecht, Belgium) photoinitiator aqueous solution at a 0.1% *w*/*v* or 0.05% *w*/*v* concentration was added dropwise, and the nanoGelMA dispersion was photocrosslinked through UV light irradiation (365 nm wavelength at 10–12 mW/cm^2^) for 2.5, 5, or 10 min. The dispersion was then centrifuged (MIKRO 220R, Andreas Hettich GmbH, Tuttlingen, Germany) at 6000 rpm and 15 °C for 30 min. The nanoGelMA particles were purified via re-dispersion in 20 mL of ddH_2_O and centrifugation (at 6000 rpm, 15 °C for 20 min). After the last re-dispersion in 15 mL of ddH_2_O, the nanoGelMA dispersion was subjected to ultrasound sonication (52 W, 20 kHz, Vibracell VCX130, Sonics, Newtown, CT, USA) for 10 min within a cold-water bath. Lastly, the nanoGelMA dispersion was filtered using 1 μm poly(tetrafluoroethylene) (PTFE) syringe filters (LLG International, Meckenheim, Germany) and used for further characterizations.

### 2.4. Characterization of nanoGelMA

#### 2.4.1. Dynamic Light Scattering (DLS) Analysis

Dynamic light scattering (DLS) analysis was performed to determine the size distribution of the nanoGelMA suspension. Specifically, the polydispersity index (PDI) and average hydrodynamic diameter (d, nm) were measured. After nanoGelMA production and purification, aliquots of the nanoGelMA dispersion were diluted and transferred into polystyrene cuvettes (Carlo Erba Reagents S.r.l., Cornaredo, Italy). Samples were analyzed at 25, 37, and 45 °C using a Zetasizer Nano S90 (Malvern Instruments, Worcestershire, United Kingdom) instrument. Before analysis, samples were equilibrated at the test temperature for 2 min. The hydrodynamic diameter and polydispersity index were calculated as the average of triplicate samples, and results were reported as the average value ± standard deviation.

#### 2.4.2. Zeta Potential Measurement

Zeta potential measurements were performed to assess the surface charge of the nanoGelMA. After nanoGelMA production and purification, aliquots of the nanoGelMA dispersion in ddH_2_O were diluted and transferred into Litesizer™ Omega cuvettes (Anton Paar Italia S.r.l., Rivoli, Italy). Samples were analyzed at 20 °C using a Litesizer™ 500 (Anton Paar Italia S.r.l., Rivoli, Italy) instrument. Zeta potential values were calculated as the average of triplicate samples on a representative nanoGelMA batch, and results were reported as the average value ± standard deviation.

#### 2.4.3. Evaluation of Production Yield

The evaluation of the nanoGelMA production yield was performed on both the first and the second GelMA desolvation steps, by collecting, freeze-drying (ALPHA 2–4 LSC, Martin Christ Gefriertrocknungsanlagen GmbH, Osterode am Harz, Germany), and weighing waste and synthesis products. Specifically, the first-step desolvation yield (HMW GelMA recovery) was calculated according to Equation (2):

(2) $$HMW\;GelMA\;recovery\left(\%\right)=\frac{w_{GelMA\_i}-w_{LMW\_GelMA}}{w_{GelMA\_i}}\times 100$$

where *w_GelMA_i_* is the initial GelMA mass (250 mg), while *w_LMW_GelMA_* is the mass of the freeze-dried low molecular weight GelMA discarded after the first GelMA desolvation.

The second-step desolvation yield was calculated by applying Equation (3):

(3) $$Second\;desolvation\;yield\left(\%\right)=\frac{w_{nanoGelMA}}{w_{HMW\_GelMA}}\times 100$$

where *w_nanoGelMA_* is the mass of the freeze-dried nanoGelMA, while *w_HMW_GelMA_* refers to the mass of high molecular weight GelMA measured as *w_HMW_GelMA_* = *w_GelMA_i_* − *w_LMW_GelMA_*.

Lastly, the overall nanoGelMA production yield was quantified according to Equation (4):

(4) $$nanoGelMA\;yield\left(\%\right)=\frac{w_{nanoGelMA}}{w_{GelMA\_i}}\times 100$$

### 2.5. Stability Test in Contact with Aqueous Environment

The nanoGelMA behavior in contact with aqueous environments was tested by incubating nanoGelMA aqueous dispersions at 37 °C (Memmert IF75, Schwabach, Germany). In detail, nanoGelMA particles were produced according to the optimized methodology and freeze-dried. The nanoGelMA dispersions at a 5 mg/mL concentration were prepared in phosphate-buffered saline (PBS, pH 7.4), phosphate buffer at an acidic pH (pH 5), and an enzymatic solution containing 5 μg/mL of Proteinase K (from Tritirachium album) (Sigma Aldrich, Milan, Italy) prepared in ddH_2_O. The initial average hydrodynamic diameter was measured through DLS analysis, before sample incubation at 37 °C. Afterwards, at predefined time points (0, 24, 48, and 96 h), samples were simply vortexed and analyzed through DLS measurements at 37 °C. Then, samples were centrifuged at 12,000 rpm and 15 °C for 5 min (Heraeus Megafuge 8R, Thermo Scientific, Osterode am Harz, Germany), resuspended in 1 mL of fresh buffers via ultrasound sonication (52 W, 20 kHz, Vibracell VCX130, Sonics, USA) for 30 s, and incubated at 37 °C again. Measurements were performed in triplicate, and results were reported as the average value ± standard deviation.

### 2.6. Preparation and Characterization of Drug-Loaded nanoGelMA

Ibuprofen (IBU, Sigma Aldrich, Milan, Italy) was selected as a hydrophobic drug to be encapsulated within the nanogel network. The IBU-loaded nanoGelMA was prepared according to the optimized method with some modifications. Briefly, 250 mg of GelMA was solubilized at a 5% *w*/*v* concentration in ddH_2_O at 45 °C, and the first desolvation was performed by adding acetone at a 1:1 *v*/*v* ratio with respect to the ddH_2_O volume. GelMA with a high molecular weight was separated and solubilized in 5 mL of ddH_2_O, and the pH of the HMW GelMA solution was adjusted to 3.5 at 45 °C using 0.1 M HCl. During the second desolvation step, 15 mL of an IBU solution in acetone was added dropwise under vigorous stirring. Then, IBU-loaded nanoGelMA particles were photocrosslinked for 10 min (at 365 nm and 10–12 mW/cm^2^) in the presence of 1 mL of LAP at 0.1% *w*/*v* as a photoinitiator. IBU concentrations of 1, 5, 25, or 50 mg/mL were reached in the final mixture. The dispersion was then centrifuged (MIKRO 220R, Andreas Hettich GmbH, Tuttlingen, Germany) at 6000 rpm and 15 °C for 30 min, and the supernatant was collected and frozen at −20 °C, after acetone evaporation under a fume hood. IBU-loaded nanoGelMA particles were purified and filtered as previously described and stored at −20 °C until use.

#### 2.6.1. Evaluation of Ibuprofen Encapsulation Efficiency

The IBU encapsulation efficiency (EE, %) was indirectly quantified by measuring the amount of unloaded IBU. In detail, at the end of IBU-loaded nanoGelMA production, the collected supernatant from the centrifugation of the dispersion was freeze-dried and analyzed using a High-Performance Liquid Chromatography (HPLC) system (Dionex Ultimate 3000 HPLC, Thermo Scientific, Waltham, MA, USA) equipped with a C18 column (5 μm, 120 Å). HPLC-grade acetonitrile (ACN, Carlo Erba Reagents S.r.l., Cornaredo, Italy) and an aqueous phosphoric acid solution (H_3_PO_4_ at 0.03% *w*/*v*) were used in a 60:40 *v*/*v* ratio as the mobile phase, at a flow rate of 1.7 mL/min, according to the IBU analysis method reported by Laurano and Boffito [29]. The freeze-dried supernatant was solubilized in 25 mL of the mobile phase and filtered using 0.45 μm poly(tetrafluoroethylene) (PTFE) syringe filters (LLG International, Meckenheim, Germany). Aliquots were analyzed at 25 °C and 214 nm for 4 min. The concentration of IBU in the supernatant samples was quantified by referring to a calibration curve based on IBU standard solutions with concentrations ranging from 0.001 mg/mL to 1 mg/mL. Lastly, the EE was calculated according to Equation (5):

(5) $$EE\left(\%\right)=\frac{w_{IBU\_i}-w_{IBU\_f}}{w_{IBU\_f}}\times 100$$

where *w_IBU_i_* is the initial mass of IBU used for IBU-loaded nanoGelMA preparation, while *w_IBU_f_* refers to the mass of IBU quantified in the supernatant. Tests were conducted in triplicate, and results were reported as the average value ± standard deviation.

#### 2.6.2. Ibuprofen Release in Physiological-like, Acidic pH, and Enzymatic Conditions

Ibuprofen release from nanoGelMA was performed under physiological-like, acidic, and enzymatic conditions. In detail, freeze-dried IBU-loaded nanoGelMA particles were resuspended at a 5 mg/mL concentration in phosphate-buffered saline (PBS, pH 7.4), phosphate buffer at an acidic pH (pH 5), and an enzymatic solution containing 5 μg/mL of Proteinase K (from Tritirachium album) (Sigma Aldrich, Milan, Italy) prepared in ddH_2_O. Samples were incubated at 37 °C (Memmert IF75, Schwabach, Germany) and centrifuged at 12,000 rpm and 15 °C for 5 min (Heraeus Megafuge 8R, Thermo Scientific, Osterode am Harz, Germany), at predefined time points (6, 24, 48, and 96 h). The release medium was collected and freeze-dried, while the IBU-loaded nanoGelMA pellets were resuspended in 1 mL of fresh buffers via ultrasound sonication (52 W, 20 kHz, Vibracell VCX130, Sonics, USA) for 30 s and incubated at 37 °C again. The release medium was analyzed through HPLC, according to the protocol described above. Briefly, each freeze-dried release medium was solubilized in 1.5 mL of the mobile phase and filtered using 0.45 μm PTFE syringe filters (LLG International, Meckenheim, Germany). Samples were analyzed at 25 °C and 214 nm for 4 min. The concentration of released IBU was quantified by referring to a calibration curve based on IBU solutions with concentrations ranging from 0.001 mg/mL to 1 mg/mL. Tests were performed in triplicate, and results were reported as the average value ± standard deviation.

### 2.7. Cytotoxicity Test

The cytocompatibility of the nanoGelMA was evaluated according to the ISO 10993:5 regulation [30], by performing the CellTiter-Blue^®^ (Promega, Milan, Italy) cell viability assay [31]. Briefly, nanoGelMA samples were prepared according to the optimized method and freeze-dried. Subsequently, they were re-dispersed in Dulbecco’s Modified Eagle Medium (DMEM, Carlo Erba Reagents S.r.l., Cornaredo, Italy) supplemented with MycoZap PLUS and 10% *v*/*v* Bovine Calf Serum (BCS, LGC Standards S.r.l., Milan, Italy) at concentrations of 5 mg/mL, 2.5 mg/mL, and 1 mg/mL. Samples were incubated at 37 °C for 24 h, and then, the supernatants were collected and filtered through a 0.22 μm filter (poly(ether sulfone) membrane, Carlo Erba Reagents S.r.l., Cornaredo, Italy). Concurrently, NIH-3T3 murine fibroblasts (ATCC^®^ CRL-1658, LGC Standards S.r.l., Milan, Italy) were cultured in complete medium (at 37 °C, 5% CO_2_, 95% humidity) and tested for mycoplasma contamination (MycoAlert PLUS mycoplasma detection kit, Lonza, Galeen, The Netherlands), before being trypsinized and seeded in a 96-well plate at 7500 cells/well. After 24 h of culture, the complete medium was substituted with 100 μL of filtered nanoGelMA extracts, and the plate was incubated again in normal culture conditions for 24 h.

To evaluate the metabolic activity of the cells, 20 μL of CellTiter-Blue^®^ solution was added to each well of the 96-well plate. Cells cultured in complete medium and treated with 2 μL of lysis agent provided by the kit were considered as negative and positive cytotoxicity controls, respectively. After 3 h of incubation, the fluorescence of reduced resazurin (resorufin) was measured using a multimodal plate reader (Perkin Elmer Victor X3, Waltham, MA, USA) at Ex/Em 535/590 nm. Cell viability was calculated by referring to the negative control. Analyses were performed in quintuplicate, and results were reported as the average value ± standard deviation.

### 2.8. Statistical Analysis

Statistical analysis was performed using GraphPad Prism 10 for Windows 10 (GraphPad Software, La Jolla, CA, USA; www.graphpad.com). Two-way ANOVA followed by Bonferroni’s multiple comparison test was conducted to compare the results. Statistical differences were assessed according to Boffito et al. [32].

## 3. Results and Discussion

### 3.1. Chemical Characterization of GelMA

To the best of our knowledge, we optimized, for the first time, a two-step desolvation procedure followed by photocrosslinking to formulate nanogels based on gelatin methacryloyl (nanoGelMA). Since the residual free amino groups of GelMA and its degree of substitution directly affect both the nanoGelMA formation and photocrosslinking, custom-made GelMA with a medium degree of methacryloylation was synthesized and characterized.

**Figure 1:**
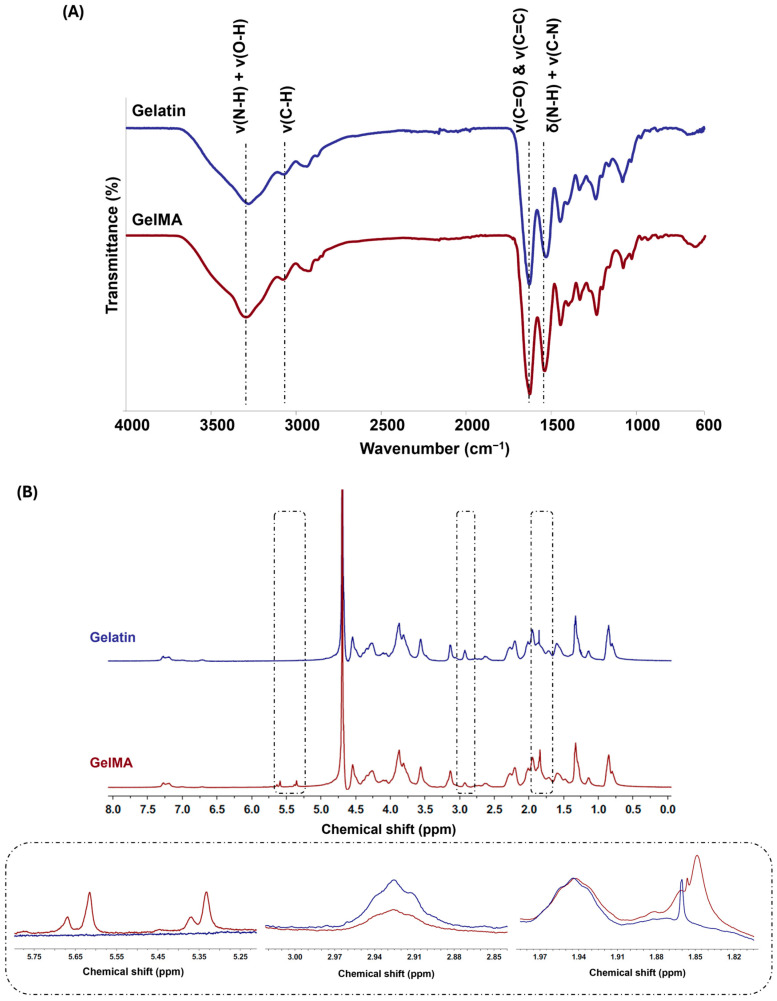
Spectra of gelatin type A from porcine skin (blue line) and GelMA (dark red line) acquired through (**A**) ATR-FTIR and (**B**) ^1^H NMR spectroscopies. Dashed lines and boxes highlight the characteristic absorption peaks and chemical shifts, respectively, of GelMA with respect to gelatin.

GelMA was synthesized through the reaction between gelatin type A from porcine skin and methacrylic anhydride (MA). A first chemical characterization was performed through ATR-FTIR spectroscopy to assess the presence of the characteristic bonds of GelMA. Figure 1A shows the ATR-FTIR spectra of gelatin type A and GelMA synthesized by adding 0.16 mL of MA per gram of gelatin. The GelMA ATR-FTIR spectrum displays the characteristic absorption peaks of gelatin, at around 3200 cm^−1^ ascribed to the O-H and N-H stretching vibrations, at ca. 3000 cm^−1^ due to the C-H stretching vibration, and at 1627 cm^−1^ and 1500 cm^−1^ related to C=O stretching vibration and the concurrent C-N stretching and N-H bending vibrations, respectively. Conversely, the characteristic peaks of methacryloyl groups turned out to be overlapped with those of native gelatin. Indeed, the C=C stretching vibration of methacryloyl groups showed its characteristic absorption peak at 1627 cm^−1^ corresponding to the amide I band of gelatin. For this reason, no further confirmation of the successful GelMA synthesis could be provided through ATR-FTIR spectroscopy. Nevertheless, the maintenance of the gelatin characteristic absorption peaks in the GelMA spectrum confirmed that the synthesis process was not detrimental to the polymer’s bulk nature.

Conversely, ^1^H NMR spectroscopic analyses proved the successful synthesis of GelMA. Indeed, compared to the gelatin spectrum, GelMA displayed a reduction in the free lysine methylene signal intensity at δ 3.0 ppm, due to the reaction between methacrylic anhydride and lysine lateral chains resulting in methacrylamide groups (Figure 1B). Furthermore, the appearance of new signals in the δ 5.4–5.7 ppm chemical shift range and at δ 1.9 ppm ascribed to the vinyl protons of methacrylamide groups and the methyl protons of methacryloyl groups, respectively, confirmed the successful GelMA synthesis [33].

The degree of methacryloylation (DoM) of GelMA was evaluated through the quantification of the residual amino groups present along the GelMA polymer chains, thus indirectly estimating the degree of substitution. In particular, the ninhydrin assay (commercially available as the Kaiser test) was performed to quantify the fraction of free amino groups [34]. The colorimetric assay revealed differences in color intensity between gelatin type A and GelMA samples, confirming the successful methacryloylation reaction that induced a reduction in the amount of free primary amines present along the GelMA polymer chains. The discrepancy in color intensity resulted in significantly different absorbance values measured through UV-Vis spectroscopic analysis between 400 and 700 nm, and GelMA DoM was estimated to be 57.5% ± 2.6% with a good synthesis repeatability among three different batches.

### 3.2. Study of nanoGelMA Formulation: Role of the Process Parameters

So far, desolvation has been widely investigated as a method to fabricate protein-based nanoparticles [35,36,37]. Among these, several studies in the literature report the use of one-step and two-step desolvation methods for the fabrication of gelatin-based nanoparticles for drug-delivery applications [38,39,40]. However, due to several issues associated with the one-step synthesis route, such as low reproducibility and poor nanoparticle quality, the two-step desolvation method is reported as the most reliable for gelatin-based nanoparticle production. In general, as first defined by Coester et al. [28], gelatin nanoparticle formation through two-step desolvation consists of several steps. In detail, a first desolvation is induced through the addition of a desolvating agent (acetone) to a gelatin aqueous solution, which allows the separation of low molecular weight and high molecular weight gelatin chains. Subsequently, the pH of the high molecular weight gelatin solution is adjusted to an acidic value to ensure the proper polymer chain repulsion via amino group protonation. Then, monodisperse nanoparticles are formed via dropwise addition of the desolvating agent under stirring. Lastly, nanoparticle stabilization is achieved by chemically crosslinking gelatin using glutaraldehyde as a crosslinking agent.

In this work, we report the formulation of nanogels based on custom-made gelatin methacryloyl through a two-step desolvation method, coupled with nanogel crosslinking through a photopolymerization reaction. Particularly, GelMA with a medium degree of methacryloylation (DoM ca. 60%) was selected as the constituent material, since it possesses both residual amino groups to be protonated and methacryloyl moieties to be photo-crosslinked, thus potentially resulting in the production of monodisperse and stable nanoGelMA. This intermediate gelatin functionalization represents a compromise to balance the nanogel formulation and subsequent network performance. Theoretically, low DoM values preserve unreacted amino groups, altering the electrostatic repulsion between the polymer chains and inducing macromolecular aggregation, while yielding poorly photo-crosslinked matrices susceptible to rapid degradation and burst drug release. Conversely, high DoM values deplete the amino groups, causing charge screening and precipitation during desolvation, while producing an over-crosslinked network with restricted swelling capacity and limited drug diffusion. A systematic study of the entire production process was performed, aiming to understand the nanogel-formation mechanism through the desolvation method and to define a reliable nanoGelMA preparation protocol. For this reason, the optimization of the nanoGelMA preparation protocol was conducted by investigating the influence of several technological parameters (volume of desolvating agent, for both the first and second desolvation procedures, pH, photoinitiator concentration, and time of the photocrosslinking phase) on nanoGelMA characteristics (average hydrodynamic diameter, polydispersity index, and production yield).

#### 3.2.1. Volume of the Desolvating Agent in the First Desolvation Step

The two-step desolvation method has been developed to produce nanoparticles with a small size and narrow size distribution. For this aim, the typical heterogeneity of the molecular weight distribution of gelatin was reduced by discarding low molecular weight gelatin through the first desolvation step [41]. The amount of desolvating agent (acetone) added during the first desolvation step was properly optimized to allow the precipitation of polymer chains with a homogeneous molecular weight. Furthermore, given that GelMA aqueous solutions exhibit an upper critical solution temperature (UCST) behavior, resulting in a sol state at temperatures above UCST [42], 45 °C was identified as the optimal temperature to perform the first step. This ensured enhanced homogeneity in the collected HMW GelMA, thereby preventing an increased recovery of material upon acetone addition due to the lower solubility of GelMA in aqueous media at lower temperatures.

**Figure 2:**
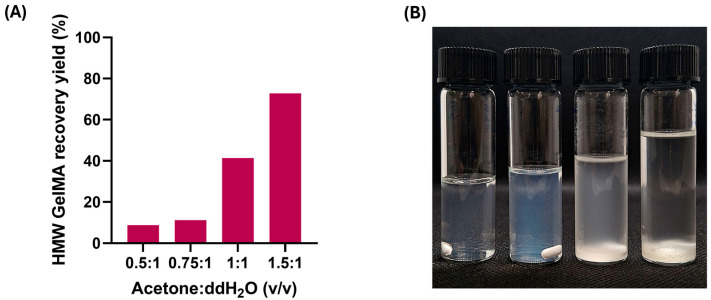
(**A**) Trend in HMW GelMA recovery as a function of the acetone:ddH_2_O volume ratio for the first desolvation step performed at 45 °C; (**B**) appearance of GelMA solutions after acetone addition at 0.5:1 *v*/*v*, 0.75:1 *v*/*v*, 1:1 *v*/*v*, and 1.5:1 *v*/*v* ratios with respect to the ddH_2_O volume used to initially solubilize GelMA (from left to right).

Different volumes of acetone were added to the GelMA aqueous solution prepared in ddH_2_O, and the first-step desolvation yield (or HMW GelMA recovery) was evaluated. As shown in Figure 2A, the HMW GelMA recovery trend was highly dependent on the volume of the desolvating agent. Indeed, as expected, the lower acetone:ddH_2_O volume ratios produced lower first-step desolvation yield values (ca. 9% and 11.2% for 0.5:1 *v*/*v* and 0.75:1 *v*/*v* ratios, respectively), due to the difficult manual separation of the two indistinct phases through decanting (Figure 2B). By increasing the acetone:ddH_2_O volume ratio to 1:1 *v*/*v*, the HMW GelMA recovery was measured to be around 45%. Consistently, a further acetone volume increase resulted in an increasing trend (HMW GelMA recovery of 72% for an acetone:ddH_2_O 1.5:1 *v*/*v* ratio). Indeed, the addition of acetone at a 1.5:1 *v*/*v* ratio with respect to the ddH_2_O volume led to the indiscriminate precipitation of all GelMA chains, which were subsequently recovered via decantation (Figure 2B).

Therefore, the optimal desolvating agent:ddH_2_O volume ratio for the first GelMA desolvation was selected as 1:1 *v*/*v*, as this condition enabled an effective separation of GelMA chains based on their molecular weight, thereby reducing the heterogeneity of the recovered fraction.

#### 3.2.2. pH Value

**Figure 3:**
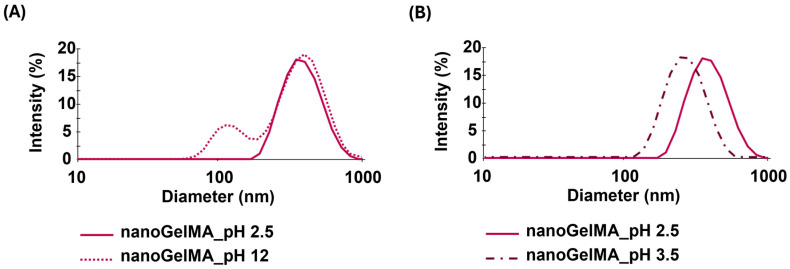
Distribution pattern (by intensity) of nanoGelMA hydrodynamic diameter measured at 25 °C for nanoGelMA formulated by setting the pH value at (**A**) 2.5 or 12 and (**B**) 2.5 or 3.5.

The pH value of the polymeric solution has been reported as one of the key parameters influencing the quality of the final product. In fact, the positive or negative net charge on gelatin induces repulsive forces between the polymer chains, thus affecting nanoparticle formation upon addition of the desolvating agent [43]. GelMA, as a derivative of gelatin, exhibits groups on the side chains that can be protonated/deprotonated by changing the environmental pH. Indeed, pH alteration results in gelatin net charge changes, due to the protonation of amino groups (i.e., NH^3+^) and deprotonation of carboxylic acid groups (i.e., COO^−^). However, due to the partial replacement of gelatin functionalities by methacryloyl moieties, the isoelectric point (pI) of GelMA varies in accordance with the degree of substitution. Particularly, as the degree of methacryloylation increases, the pI value decreases accordingly [44]. To understand the role exerted by the pH value of the HMW GelMA solution on nanoGelMA formation, three pH conditions (pH 2.5, pH 12, and no pH adjustment) were initially evaluated. The condition of no pH adjustment (ca. pH 6) resulted in almost no nanogel formation, since the final product was prevalently formed by other species, such as filaments. This preliminary result was in agreement with the literature that has already highlighted the pivotal role exerted by electrostatic interactions in determining gelatin-based nanoparticle formation. Indeed, it was reported that gelatin nanoparticles prepared at a pH value close to the pI exhibited larger sizes and instability due to aggregation of gelatin molecules, while the high surface charge of the polymer chains guaranteed greater repulsive forces to prevent these phenomena [45]. Conversely, nanoGelMA prepared by adjusting the pH to 2.5 or 12 was further characterized through DLS analysis (samples coded as nanoGelMA_pH 2.5 and nanoGelMA_pH 12, respectively). Figure 3A reports the intensity distribution profiles of the nanoGelMA hydrodynamic diameter for the two tested conditions. The nanoGelMA prepared from the alkaline HMW GelMA solution (nanoGelMA_pH 12) exhibited a broad and bimodal hydrodynamic diameter distribution (PDI of 0.51 ± 0.03). Conversely, the nanoGelMA prepared at an acidic pH showed a Gaussian distribution, a hydrodynamic diameter of 304.3 ± 9.2 nm, and a PDI of 0.37 ± 0.04. Hence, the acidic pH condition was selected for additional investigations. In detail, three different acidic pH conditions were tested, ranging between pH 2.5 and 4.5. Similarly to the no pH adjustment condition, setting the pH at 4.5 (coded as nanoGelMA_pH 4.5) resulted in almost no nanogel formation. As reported in Table 1, this observation was also confirmed by the nanoGelMA production yield that resulted to be lower than 1%. Conversely, nanoGelMA samples formulated at pH 2.5 and 3.5 (referred to as nanoGelMA_pH 2.5 and nanoGelMA_pH 3.5, respectively) showed a comparable second desolvation yield (ca. 65%) and, accordingly, a nanoGelMA production yield equal to approx. 26%. Thus, the nanoGelMA prepared by setting lower acidic pH values was further characterized via DLS analysis. Figure 3B shows the distribution pattern by intensity of nanoGelMA_pH 2.5 compared to nanoGelMA_pH 3.5, with the former showing a shift toward a higher nanogel size, as also confirmed by the average hydrodynamic diameter and polydispersity index measurements, summarized in Table 1. nanoGelMA_pH 3.5 possessed a smaller hydrodynamic diameter compared to nanoGelMA_pH 2.5 (i.e., 251.9 ± 11 vs. 304.3 ± 9.2 nm, *p* < 0.01) and a narrower size distribution (i.e., PDI 0.22 ± 0.01 vs. 0.37 ± 0.04, *p* < 0.01). Interestingly, the optimal HMW GelMA solution pH value resulted to be 3.5, instead of pH 2.5, which is generally reported as the optimal pH for preparing gelatin-based nanoparticles [28]. As expected, due to the medium substitution degree of GelMA, the maximum protonation of residual amino groups was achieved at a higher pH value compared to native gelatin, and a further decrease in pH (at 2.5) negatively affected the properties of the resulting nanoGelMA. This observation suggested that the addition of an excessive volume of hydrochloric acid (0.1 M HCl) to adjust the HMW GelMA solution pH to pH 2.5 resulted in an increase in the number of anions (i.e., Cl^−^) present in the reaction mixture, which consequently induced a reduction of the intermolecular electrostatic repulsion between charged GelMA chains. This effect, also known as Debye–Hückel screening, can occur when nanoparticles interact with electrolytes and the localized ion concentration shields the surface charge of the polymer chains [46].

Therefore, pH 3.5 was selected as the optimal pH value for the HMW GelMA aqueous solution, to be subjected to the second desolvation and nanoGelMA formation. Indeed, this pH value ensured the optimal electrostatic interaction among GelMA polymer chains.

#### 3.2.3. Volume of the Desolvating Agent in the Second Desolvation Step

**Figure 4:**
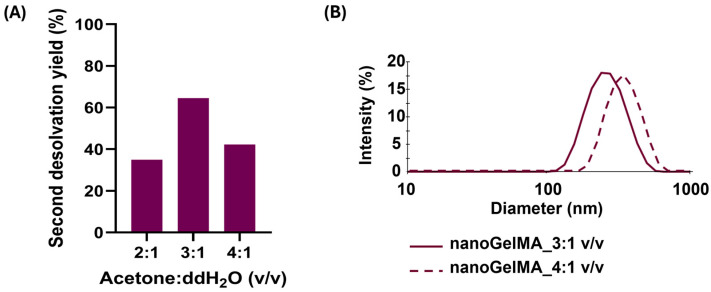
(**A**) Trend of the second desolvation yield (%) as a function of the volume of desolvating agent added at 2:1 *v*/*v*, 3:1 *v*/*v*, and 4:1 *v*/*v* ratios with respect to the ddH_2_O volume used to prepare the starting HMW GelMA solution; (**B**) distribution pattern (by intensity) of nanoGelMA hydrodynamic diameter measured at 25 °C for nanoGelMA formulated via acetone addition at 3:1 *v*/*v* and 4:1 *v*/*v* ratios.

In general, the second step of the desolvation process determines HMW GelMA self-assembly into nanoparticles, due to the dropwise addition of the desolvating agent under vigorous stirring. The formation of the nanoGelMA was investigated upon acetone addition to the HMW GelMA solution (pH adjusted to 3.5) at three different volume ratios, i.e., 2:1 *v*/*v*, 3:1 *v*/*v*, and 4:1 *v*/*v* ratios, with respect to the ddH_2_O volume used to prepare the HMW GelMA solution (sample coded as nanoGelMA_2:1 *v*/*v*, nanoGelMA_3:1 *v*/*v*, and nanoGelMA_4:1 *v*/*v*, respectively). Figure 4A displays the second-step desolvation yield trend as a function of acetone:ddH_2_O volume ratio. By increasing the amount of acetone from a 2:1 to 3:1 *v*/*v* ratio, the second desolvation yield increased accordingly (i.e., from 35% to 64.5%). This trend could be explained by considering the mechanism underpinning nanogel formation occurring during the second step of the desolvation process. As proposed by Hassani Besheli et al., two main phases could be distinguished upon gradual acetone addition, visually identified by an increasing solution turbidity [47]. Specifically, the initial addition of acetone led to the dehydration of GelMA, and the localized supersaturation resulted in polymer chains collapsing into small nanoparticles, due to the reduction of electrostatic repulsion between charged moieties along the GelMA backbone (i.e., protonated -NH_2_ at pH 3.5). Due to the small nanoparticles size, no changes in solution turbidity were observed at this stage. Nevertheless, the incorporation of additional desolvating agent into the reaction mixture triggered the growth phase, characterized by the self-assembly of primary small nanosystems into more complex spherical nanostructures. The formation of these latter nanostructures was probably disfavored at an acetone:ddH_2_O 2:1 *v*/*v* ratio, as it was insufficient to reach the growth phase equilibrium. As a result, only a few homogeneous nanogels were crosslinked and collected (d 307 ± 4.4 nm and PDI 0.2 ± 0.01). Interestingly, a reverse trend was observed upon the addition of acetone at 4:1 *v*/*v* ratio, with a second desolvation yield decreasing to 42.3% (compared to 64.5% for nanoGelMA_3:1 *v*/*v*). In this case, larger nanoGelMA and aggregates likely developed and were discarded during nanogel purification and filtration. Indeed, this outcome was confirmed by the hydrodynamic diameter distribution patterns (by intensity) measured via DLS analyses; the hydrodynamic diameter distribution of nanoGelMA_4:1 *v*/*v* shifted toward larger sizes compared to nanoGelMA_3:1 *v*/*v* (d 251.9 ± 11.0 nm and PDI 0.22 ± 0.01 vs. d 330 ± 5.7 nm and PDI 0.1 ± 0.01) (Figure 4B).

Therefore, acetone at a 3:1 *v*/*v* ratio was fixed as the optimal condition for the second GelMA desolvation. This set-up indeed represents the optimal condition in terms of both the production yield and the final dimensions of the nanostructures.

#### 3.2.4. nanoGelMA Photocrosslinking Parameters

**Figure 5:**
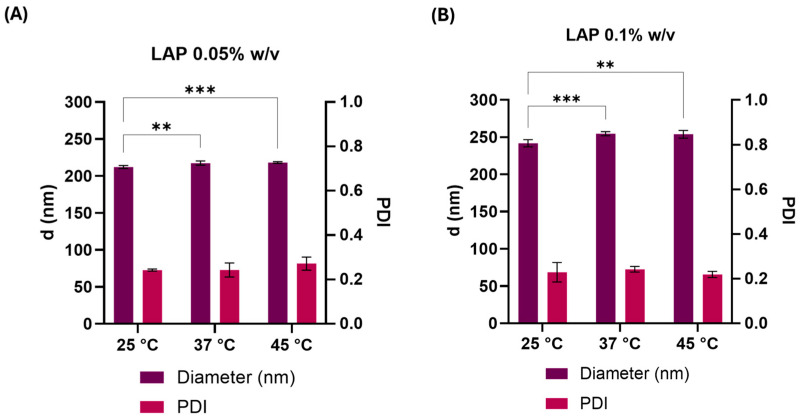
Average hydrodynamic diameter (d, nm) and polydispersity index (PDI) values measured at different temperatures (i.e., 25, 37, 45 °C) for nanoGelMA photo-cured at 365 nm and 10–12 mW/cm^2^ for 10 min, in the presence of LAP at a (**A**) 0.05% *w*/*v* and (**B**) 0.1% *w*/*v* concentration. ** *p* < 0.01, *** *p* < 0.001.

To stabilize the GelMA-based nanoparticles, the photo-crosslinking of the methacryloyl moieties exposed along the GelMA backbone, in the presence of a photo-initiator and upon UV-light irradiation, was exploited [48]. In particular, the photo-crosslinking phase was optimized in terms of the LAP concentration and UV-light exposure time. Figure 5 reports the average hydrodynamic diameter and polydispersity index values of nanoGelMA photocrosslinked through UV-light exposure at 365 nm and 10–12 mW/cm^2^ for 10 min in the presence of LAP at a 0.05% *w*/*v* (Figure 5A) or 0.1% *w*/*v* (Figure 5B) concentration (samples referred to as nanoGelMA_LAP 0.05% and nanoGelMA_LAP 0.1%, respectively). Both the tested LAP concentrations resulted in stable photo-crosslinked nanogels across the different analyzed temperatures. Irrespective of the LAP content, a slight increase in the nanoGelMA size was observed as the analysis temperature increased from 25 °C to 37 °C. For instance, nanoGelMA_LAP 0.1% increased its hydrodynamic diameter from 241.7 ± 4.7 nm at 25 °C to 254 ± 5.2 nm at 37 °C (*p* = 0.0008). Interestingly, photo-crosslinking performed in the presence of a low photo-initiator concentration (i.e., 0.05% *w*/*v*) resulted in nanoGelMA with a smaller size (at 25 °C, average hydrodynamic diameter of 212 ± 2 nm for nanoGelMA_LAP 0.05% vs. 241.7 ± 4.7 nm for nanoGelMA_LAP 0.1%, *p* = 0.0006) and PDI of approx. 0.2. However, the evaluation of the nanoGelMA production yield (Table 2), and specifically the second-desolvation yield (i.e., 40.3% for nanoGelMA_LAP 0.05% vs. 64.1% for nanoGelMA_LAP 0.1%), evidenced a lower nanoGelMA recovery at a 0.05% *w*/*v* LAP concentration compared to 0.1% *w*/*v*. This result could likely be attributed to the incomplete nanoGelMA stabilization achieved at a 0.05% *w*/*v* LAP content, which led to the presence of uncrosslinked or poorly crosslinked nanogels that were discarded during the purification steps. Based on these results, a LAP concentration of 0.1% *w*/*v* was selected as optimal for nanoGelMA stabilization through photo-crosslinking.

**Figure 6:**
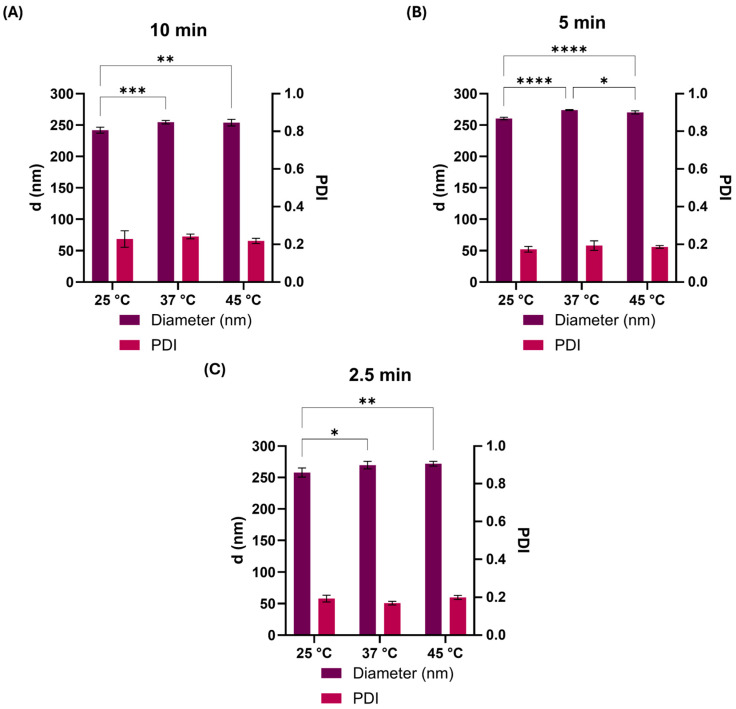
Average hydrodynamic diameter (d, nm) and polydispersity index (PDI) values measured at different temperatures (i.e., 25, 37, 45 °C) for nanoGelMA photo-cured at 365 nm and 10–12 mW/cm^2^, in the presence of LAP at a 0.1% *w*/*v* concentration, for (**A**) 10 min, (**B**) 5 min, and (**C**) 2.5 min. * *p* < 0.05, ** *p* < 0.01, *** *p* < 0.001, **** *p* < 0.0001.

The reduction in UV-light exposure time was evaluated by performing the nanoGelMA photo-crosslinking for 10, 5, or 2.5 min in the presence of the selected LAP concentration (i.e., 0.1% *w*/*v*) (samples coded as nanoGelMA_UV 10 min, nanoGelMA_UV 5 min, and nanoGelMA_UV 2.5 min, respectively). All the tested UV-light irradiation timings ensured the successful production of a stable nanoGelMA, whose average hydrodynamic diameter remained approximately constant as the temperature increased up to 45 °C, as shown in Figure 6. For instance, the hydrodynamic diameter of nanoGelMA_UV 2.5 min increased from 258 ± 7.2 nm at 25 °C to 272 ± 3.7 nm at 45 °C (*p* < 0.05). Longer UV-light exposure times resulted in smaller nanoGelMA sizes (Figure 6A). In fact, at 25 °C, the hydrodynamic diameter of nanoGelMA photo-cured for 5 (Figure 6B) or 2.5 min (Figure 6C) was measured to be 260 ± 2.0 nm and 258 ± 7.2 nm, respectively, compared to 241.7 ± 4.7 nm for nanoGelMA_UV 10 min (UV exposure 10 min vs. 5 min *p* < 0.01, 10 min vs. 2.5 min *p* < 0.05). Furthermore, similar to the trends observed when varying the photo-initiator concentration, as the photo-curing time decreased, the nanoGelMA yield decreased accordingly. Indeed, lower second desolvation yields were recorded for 5 and 2.5 min of nanoGelMA UV-light irradiation (i.e., 50.8% and 40%, respectively, compared to 64.1% for nanoGelMA_UV 10 min), resulting in a lower overall production yield, as summarized in Table 3. UV-light irradiation for less than 10 min likely produced a heterogeneous final product composed of partially photo-crosslinked nanogels, which were discarded during purification.

**Figure 7:**
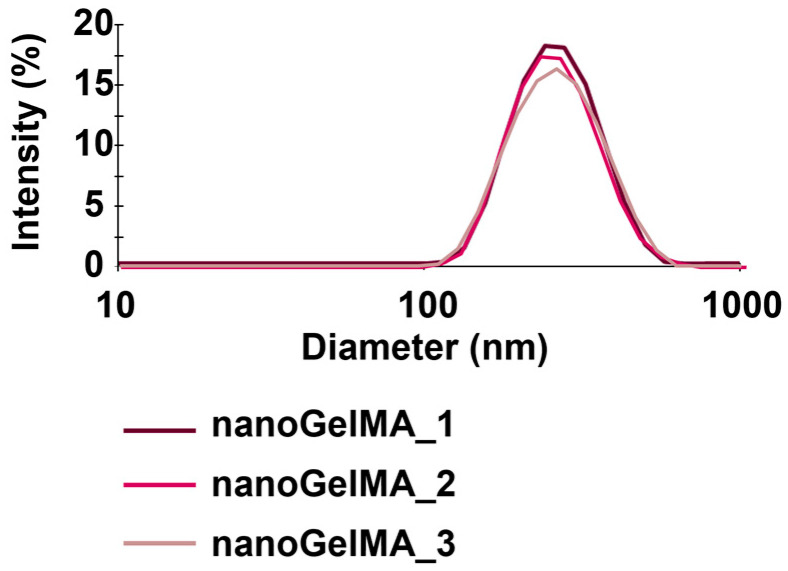
Distribution pattern (by intensity) of nanoGelMA hydrodynamic diameter measured at 25 °C for three independent nanoGelMA batches formulated according to the optimized protocol.

Therefore, photo-crosslinking via UV-light exposure for 10 min in the presence of LAP at a 0.1% *w*/*v* concentration was identified as the optimal condition to collect a high yield of stable nanoGelMA. According to the optimized protocol summarized in Table 4, nanoGelMA was produced with an average hydrodynamic diameter of 249.5 ± 9.7 nm, a polydispersity index of 0.21 ± 0.02, and a production yield of 26.5% ± 1.3%. The highly overlapping size distribution profiles confirmed good repeatability among three different batches (Figure 7). Furthermore, the zeta potential of nanoGelMA in ddH_2_O, measured in triplicate on a representative batch, was −12.4 ± 1.8 mV (Figure S1).

### 3.3. Evaluation of nanoGelMA Physico-Chemical Stability in Aqueous Environments

**Figure 8:**
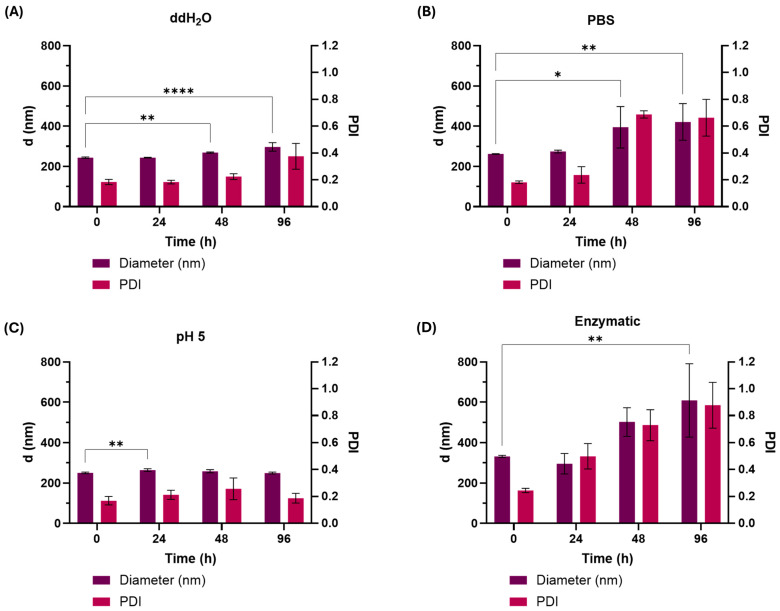
Average hydrodynamic diameter (d, nm) and polydispersity index (PDI) measured at different time points (i.e., for as-prepared samples -t0-, and after 24, 48, and 96 h of incubation at 37 °C) and 37 °C for nanoGelMA dispersions prepared in (**A**) double-distilled water (ddH_2_O), (**B**) phosphate-buffered saline solution (PBS, pH 7.4), (**C**) phosphate buffer at pH 5, and (**D**) enzymatic solution containing 5 μg/mL of Proteinase K. * *p* < 0.05, ** *p* < 0.01, **** *p* < 0.0001.

The stability of nanoGelMA in contact with aqueous environments was evaluated by dispersing the nanogels in different media at 37 °C for up to 4 days. Figure 8 reports the average hydrodynamic diameter and polydispersity index measured at different time points for nanoGelMA dispersions prepared in ddH_2_O, phosphate-buffered saline solution (PBS, pH 7.4), phosphate buffer at pH 5, and an enzymatic solution containing 5 μg/mL of Proteinase K. The registered trends evidenced a clear dependence of nanogel stability on the dispersion medium. In ddH_2_O (Figure 8A), the nanogels exhibited a slight increase in the hydrodynamic diameter (i.e., ca. 240 nm at t0 vs. 270 and 300 nm at 48 h and 96 h, respectively), while the PDI values remained constant at around 0.2 over time. Indeed, the low ionic strength of the aqueous environment resulted in reduced swelling and aggregation phenomena, indicating that nanoGelMA remained overall highly stable when dispersed in ddH_2_O. Conversely, the presence of salt in PBS increased the ionic strength, which could screen electrostatic repulsion, interfere with hydrogen bonding within the nanoGelMA network, and promote osmotic swelling [49,50,51], thus leading to an increase in both the diameter and PDI and slight aggregation (Figure 8B). Indeed, from the early stage of incubation, nanoGelMA dispersion in PBS showed a higher average hydrodynamic diameter compared to the same nanogel batch resuspended in ddH_2_O (i.e., 260 nm vs. 240 nm at t0) and exhibited a more pronounced increase in diameter over time, rising from ca. 260 nm at t0 to 400 nm at 48 h and 420 nm at 96 h. This trend was also confirmed by an increase in PDI up to 0.6 at 4 days of incubation, suggesting a reduction in homogeneity. The nanoGelMA dispersed in phosphate buffer at pH 5 (Figure 8C) showed remarkable stability, with the hydrodynamic diameter and PDI remaining nearly constant (i.e., average hydrodynamic diameter of 260 nm and PDI of 0.2). This behavior suggested that the acidic conditions did not significantly affect the structural integrity of the nanoGelMA network. This stability could be attributed to the concurrent protonation of the residual amino groups and the neutralization of carboxylic acid groups under acidic conditions, resulting in a reduction in effective electrostatic interactions within the network. As expected, Proteinase K induced the rapid degradation of nanoGelMA. Indeed, the average hydrodynamic diameter increased from 330 nm at t0 to approximately 500 and 600 nm at 48 h and 96 h, respectively, while the PDI increased to a value close to 1 (Figure 8D). The observed broadening of the particle size distribution was attributed to network loosening and swelling, followed by a reduction in nanogel mass due to progressive degradation [52,53]. Overall, differences in stability behavior could be attributed to the nanoGelMA network structure, the effect of the ionic environment on swelling and aggregation, the pH-dependent charge of gelatin functionalities, and the susceptibility of the gelatin backbone to enzymes, highlighting the responsiveness of nanoGelMA to biologically relevant environments.

### 3.4. Cytotoxicity Evaluation

**Figure 9:**
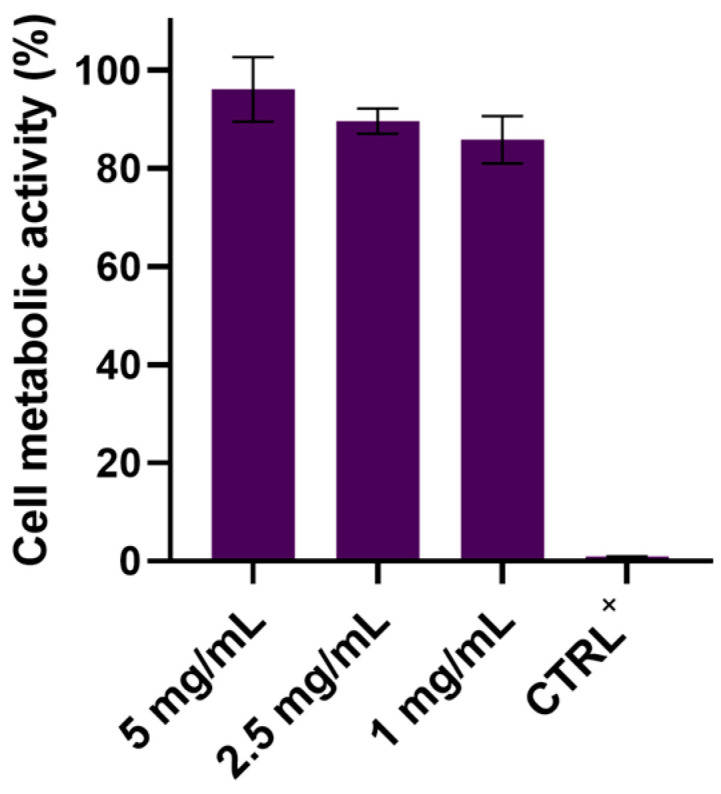
Cell metabolic activity (%) of NIH-3T3 cells in contact with extracts of nanoGelMA at 5, 2.5, and 1 mg/mL. Lysed cells were used as the positive cytotoxicity control (CTRL^+^).

The cytocompatibility of nanoGelMA was preliminarily evaluated in accordance with ISO 10993-5 guidelines [30]. The CellTiter-Blue^®^ assay demonstrated a high level of metabolic activity in NIH-3T3 murine fibroblasts exposed to nanoGelMA eluates, irrespective of the tested concentration. In fact, as reported in Figure 9, the cell metabolic activity of NIH-3T3 cells was measured to be ca. 96%, 90%, and 86% for cells cultured for 24 h in contact with extracts of nanoGelMA at 5, 2.5, and 1 mg/mL, respectively, with no statistically significant differences observed. Based on the regulatory cytocompatibility threshold (cell viability ≥ 70%), this biological evaluation confirmed the cytocompatibility of the nanoGelMA. These preliminary findings indicate that the developed nanocarriers possess promising biocompatibility, attributed to the use of gelatin, a natural polymer, as a precursor, which effectively supported cell viability. Nevertheless, since the cytocompatibility evaluation was restricted to short-term (i.e., 24 h), indirect extract testing on a standard murine fibroblast line, further investigations are required to thoroughly assess the nanoGelMA behavior in a more complex biological environment.

### 3.5. Ibuprofen-Loaded nanoGelMA: Encapsulation Efficiency and Release Study

**Figure 10:**
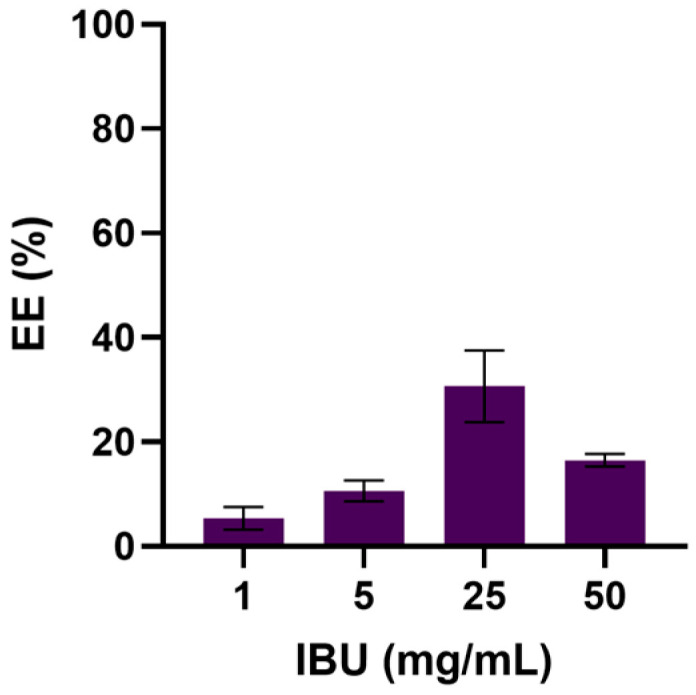
Trend of encapsulation efficiency (EE %) as a function of the initial concentration of ibuprofen (i.e., 1, 5, 25, and 50 mg/mL) added during IBU-loaded nanoGelMA preparation.

The potential of nanoGelMA as a drug delivery system was assessed by investigating the encapsulation and release of ibuprofen (IBU) as a model anti-inflammatory drug. IBU-loaded nanoGelMA particles were formulated via direct drug encapsulation during nanogel formation, following a slightly modified preparation protocol. Specifically, the successful formulation of IBU-loaded nanoGelMA was achieved by adding an IBU solution in acetone dropwise during the second desolvation step, resulting in simultaneous nanogel formation and drug loading. The encapsulation efficiency (EE, %) was optimized by varying the initial drug amount added during nanogel preparation. As shown in Figure 10, the encapsulation efficiency increased with an increasing drug concentration, reaching a maximum of 30.6% ± 6.8% at an initial IBU concentration of 25 mg/mL. Above this optimal value, a slight decrease was observed, indicating saturation of the available binding and trapping sites within the nanoGelMA network. Therefore, the loading capacity of the polymer matrix is limited, resulting in ineffective drug encapsulation and the removal of excess drug during the purification step. The observed encapsulation behavior can be attributed to a combination of weak interactions between drug molecules and polymer chains forming the nanoGelMA network and drug precipitation during the desolvation step. In particular, IBU is a low molecular weight hydrophobic molecule, and its incorporation relies primarily on physical entrapment within the hydrophilic nanoGelMA network, which may further limit loading efficiency. The encapsulation efficiency achieved in this study is within the wide range reported in the literature for gelatin- and GelMA-based drug delivery systems, which strongly depends on the physico-chemical properties of the payload, the formulation method, and the processing conditions used to produce the micro- and nano-carriers [54,55,56]. Overall, these findings support the suitability of the nanoGelMA as a drug delivery platform and provide a basis for the further optimization of drug-loading performance.

**Figure 11:**
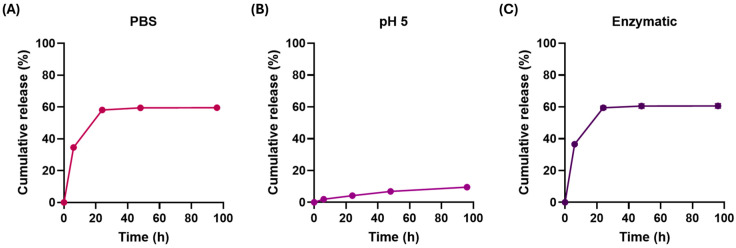
IBU release profile from IBU-loaded nanoGelMA dispersion in (**A**) phosphate-buffered saline solution (PBS, pH 7.4), (**B**) phosphate buffer at pH 5, and (**C**) enzymatic solution containing 5 μg/mL of Proteinase K.

IBU release was studied in vitro under different environmental conditions (i.e., in phosphate-buffered saline solution, PBS at pH 7.4, phosphate buffer at pH 5, and in the presence of Proteinase K at 5 μg/mL) for 4 days. The release profiles demonstrated a close correlation with the stability behavior of the nanoGelMA under the same conditions. Indeed, in PBS (Figure 11A), a rapid initial release of 34.5% ± 0.7% in 6 h and 57.6% ± 2% in 24 h was observed, followed by a plateau. This result was consistent with the swelling and partial structural relaxation of the nanoGelMA network due to the high ionic strength of the buffer, which facilitated IBU diffusion. In contrast, at pH 5 (Figure 11B), drug release was significantly slower and more sustained over time, reaching only ca. 10% after 96 h. The release kinetics were in strong agreement with the high stability and limited swelling observed under acidic conditions, indicating restricted drug diffusion through the nanoGelMA network. Furthermore, the occurrence of weak interactions between the entrapped payload and the nanoGelMA network (given that at pH 5 IBU is partially deprotonated and negatively charged, with a pK_a_ equal to 4.59) [57], could additionally limit molecule release. Under enzymatic conditions (Figure 11C), a rapid IBU release of 61% ± 7% within 24 h was quantified, directly correlating with the degradation of nanoGelMA triggered by Proteinase K. Consequently, the enzymatic cleavage of peptide bonds accelerated drug release through concurrent drug diffusion and matrix degradation. Overall, these results highlight that the drug-loaded nanoGelMA exhibits environment-responsive release, with release kinetics and mechanisms modulated by the surrounding environment, particularly in the presence of ions, pH changes, or enzymatic activity.

## 4. Conclusions

In this work, GelMA-based nanogels (nanoGelMA) were successfully developed through a combined two-step desolvation and photo-crosslinking method. The systematic optimization of formulation parameters (i.e., amount of desolvating agent, pH, photoinitiator concentration, and photocuring time) led to the identification of optimal conditions, enabling the production of nano-scale hydrogels with a controlled size, narrow size distribution, and a good production yield. The designed nanoGelMA exhibited high stability in aqueous and acidic environments while showing pronounced swelling under physiological-like conditions and susceptibility to enzymatic degradation, highlighting its responsiveness to external stimuli. Furthermore, the nanoGelMA displayed excellent cytocompatibility across the tested concentrations, demonstrating its potential for biomedical applications. The successful encapsulation of ibuprofen during nanogel formation and the environment-responsive release behavior, modulated by the ionic strength, pH, and enzymatic activity, further confirmed the suitability of the nanoGelMA as a drug delivery system. Subsequent biological validations should be conducted under more physiologically relevant conditions, alongside the functional evaluation of the drug-loaded nanogels. In conclusion, the designed nanoGelMA represents a promising and versatile nanocarrier platform for the development of advanced, stimuli-responsive drug delivery strategies for site-specific anti-inflammatory therapies and other precision medicine approaches, including drug delivery to tissues characterized by altered pH or enzymatic activity (e.g., infected tissues, tumor microenvironments).

## Figures and Tables

**Table 1:** Average hydrodynamic diameter (nm) and polydispersity index (PDI) measured at 25 °C, and production yield (%) of nanoGelMA formulated by setting the pH of the HMW GelMA solution at 2.5, 3.5, or 4.5.

	Average Hydrodynamic Diameter (nm)	PDI	nanoGelMA Production Yield (%)
nanoGelMA_pH 2.5	304.3 ± 9.2	0.37 ± 0.004	25.8
nanoGelMA_pH 3.5	251.9 ± 11.0	0.22 ± 0.01	26.7
nanoGelMA_pH 4.5	- ^#^	- ^#^	0.9

^#^ not calculated data.

**Table 2:** Average hydrodynamic diameter (nm) and polydispersity index (PDI) measured at 25 °C, and production yield (%) of nanoGelMA photo-crosslinked at 365 nm and 10–12 mW/cm^2^ for 10 min, in the presence of LAP photo-initiator at a 0.05% *w*/*v* or 0.1% *w*/*v* concentration.

	Average Hydrodynamic Diameter (nm)	PDI	nanoGelMA Production Yield (%)
nanoGelMA_LAP 0.05%	212.0 ± 2.0	0.24 ± 0.004	16.5
nanoGelMA_LAP 0.1%	241.7 ± 4.7	0.23 ± 0.04	26.7

**Table 3:** Average hydrodynamic diameter (nm) and polydispersity index (PDI) measured at 25 °C, and production yield (%) of nanoGelMA photo-crosslinked at 365 nm and 10–12 mW/cm^2^, in the presence of LAP photo-initiator at a 0.1% *w*/*v* concentration, for 10 min, 5 min, or 2.5 min.

	Average Hydrodynamic Diameter (nm)	PDI	nanoGelMA Production Yield (%)
nanoGelMA_UV 10 min	241.7 ± 4.7	0.23 ± 0.04	26.7
nanoGelMA_UV 5 min	260.0 ± 2.0	0.17 ± 0.01	18.5
nanoGelMA_UV 2.5 min	258.0 ± 7.2	0.19 ± 0.01	16.2

**Table 4:** Summary of the process parameter optimization for nanoGelMA formulation. Comparison among all the tested conditions in terms of average hydrodynamic diameter (nm), polydispersity index (PDI) measured at 25 °C, and process yield (%), including reasons for the selection (S) or discarding (D) of each tested condition.

Process Parameter	Tested Conditions	Average Hydrodynamic Diameter (nm)	PDI	Yield (%)	Reasons for Selection (S)/Discarding (D)
				First desolvation	
First desolvation acetone:ddH_2_O volume ratio	0.5:1	- ^#^	- ^#^	8.8	D: Poor phase separation
	0.75:1	- ^#^	- ^#^	11.2	D: Insufficient recovery yield
	1:1	- ^#^	- ^#^	41.4	S: Effective GelMA separation
	1.5:1	- ^#^	- ^#^	72.8	D: Indiscriminate GelMA precipitation
				nanoGelMA production	
pH	2.5	304.3 ± 9.2	0.37 ± 0.004	25.8	D: Surplus of ions causing screening effect
	3.5	251.9 ± 11.0	0.22 ± 0.01	26.7	S: Optimal electrostatic interactions
	4.5	- ^#^	- ^#^	0.9	D: No nanogel formation (other species)
	12	292.7 ± 11.3	0.51 ± 0.03	- ^#^	D: Broad and bimodal diameter distribution
				Second desolvation	
Second desolvation acetone:ddH_2_O volume ratio	2:1	307.0 ± 4.4	0.20 ± 0.01	34.9	D: Unfavorable nanogel growth phase
	3:1	251.9 ± 11.0	0.22 ± 0.01	64.6	S: Equilibrium between GelMA dehydration and nanogel growth phase
	4:1	330.0 ± 5.7	0.10 ± 0.01	42.3	D: Larger nanoGelMA and aggregates
				nanoGelMA production	
Photoinitiator (% *w*/*v*)	0.05	212.0 ± 2.0	0.24 ± 0.004	16.5	D: Incomplete photo-crosslinking
	0.1	241.7 ± 4.7	0.23 ± 0.04	26.7	S: Effective nanoGelMA stabilization
UV-exposure time (minutes)	10	241.7 ± 4.7	0.23 ± 0.04	26.7	S: Complete network stabilization
	5	260.0 ± 2.0	0.17 ± 0.01	18.5	D: Incomplete photo-crosslinking
	2.5	258.0 ± 7.2	0.19 ± 0.01	16.2	D: Incomplete photo-crosslinking

^#^ not calculated data.

## Data Availability

The original contributions presented in this study are included in the article/Supplementary Material. Further inquiries can be directed to the corresponding author.

## Supplementary Materials

The following supporting information can be downloaded at: https://www.mdpi.com/article/10.3390/pharmaceutics18070812/s1, Table S1: Comparison of gelatin- and gelatin methacryloyl (GelMA)-based nanoparticle systems reported in the literature with respect to constituent material, preparation method, crosslinking strategy, and particle size; Figure S1: Average Zeta potential distribution measured on a representative batch of nanoGelMA dispersion in ddH_2_O.

## References

[1] Soni K.S., Desale S.S., Bronich T.K. (2016). Nanogels: An overview of properties, biomedical applications and obstacles to clinical translation. J. Control. Release.

[2] Yin Y., Hu B., Yuan X., Cai L., Gao H., Yang Q. (2020). Nanogel: A versatile nano-delivery system for biomedical applications. Pharmaceutics.

[3] De Lima C.S.A., Balogh T.S., Varca J.P.R.O., Varca G.H.C., Lug A.B., Camacho-cruz L.A., Bucio E., Kadlubowski S.S. (2020). An Updated Review of Macro, Micro, and Nanostructured Hydrogels for Biomedical and Pharmaceutical Applications. Phamarceutics.

[4] Oluwatoyin Shoge M. (2023). Applications of Nanogel in Drug Delivery. Hydrogels and Nanogels-Applications in Medicine.

[5] Vetriselvan S., Fuloria N.K., Chinni S.V., Sekar M., Fuloria S., Celia C. (2023). Nanogels as novel drug nanocarriers for CNS drug delivery. Front. Mol. Biosci..

[6] Chander S., Kulkarni G.T., Dhiman N., Kharkwal H. (2021). Protein-Based Nanohydrogels for Bioactive Delivery. Front. Chem..

[7] Altuntaş E., Özkan B., Güngör S., Özsoy Y. (2023). Biopolymer-Based Nanogel Approach in Drug Delivery: Basic Concept and Current Developments. Pharmaceutics.

[8] Elzoghby A.O. (2013). Gelatin-based nanoparticles as drug and gene delivery systems: Reviewing three decades of research. J. Control. Release.

[9] Khan S.A. (2020). Opportunities and challenges in the techniques used for preparation of gelatin nanoparticles. Pak. J. Pharm. Sci..

[10] Carvalho J.A., Abreu A.S., Ferreira V.T.P., Gonçalves E.P., Tedesco A.C., Pinto J.G., Ferreira-Strixino J., Beltrame Junior M., Simioni A.R. (2018). Preparation of gelatin nanoparticles by two step desolvation method for application in photodynamic therapy. J. Biomater. Sci. Polym. Ed..

[11] Ballantyne B., Jordan S.L. (2001). Toxicological, medical and industrial hygiene aspects of glutaraldehyde with particular reference to its biocidal use in cold sterilization procedures. J. Appl. Toxicol..

[12] Park C., Vo C.L.N., Kang T., Oh E., Lee B.J. (2015). New method and characterization of self-assembled gelatin-oleic nanoparticles using a desolvation method via carbodiimide/N-hydroxysuccinimide (EDC/NHS) reaction. Eur. J. Pharm. Biopharm..

[13] Cascone M.G., Lazzeri L., Carmignani C., Zhu Z. (2002). Gelatin nanoparticles produced by a simple W/O emulsion as delivery system for methotrexate. J. Mater. Sci. Mater. Med..

[14] Kim J., Gauvin R., Yoon H.J., Kim J.H., Kwon S.M., Park H.J., Baek S.H., Cha J.M., Bae H. (2016). Skin penetration-inducing gelatin methacryloyl nanogels for transdermal macromolecule delivery. Macromol. Res..

[15] Choubey J., Bajpai A.K. (2010). Investigation on magnetically controlled delivery of doxorubicin from superparamagnetic nanocarriers of gelatin crosslinked with genipin. J. Mater. Sci. Mater. Med..

[16] Khan S.A., Schneider M. (2013). Nanoprecipitation versus two step desolvation technique for the preparation of gelatin nanoparticles. Colloid. Nanocrystals Biomed. Appl. VIII.

[17] Lee E.J., Khan S.A., Lim K.-H. (2011). Gelatin Nanoparticle Preparation by Nanoprecipitation. J. Biomater. Sci. Polym. Ed..

[18] Narayani R., Rao K.P. (1995). pH-responsive gelatin microspheres for oral delivery of anticancer drug methotrexate. J. Appl. Polym. Sci..

[19] Gunji S., Obama K., Matsui M. (2013). A novel drug delivery system of intraperitoneal chemotherapy for peritoneal carcinomatosis using gelatin microspheres incorporating cisplatin. Surgery.

[20] Fan C., Wang D. (2016). Novel Gelatin-based Nano-gels with Coordination-induced Drug Loading for Intracellular Delivery. J. Mater. Sci. Technol..

[21] Jiang X., Du Z., Zhang X., Zaman F. (2023). Gelatin-based anticancer drug delivery nanosystems: A mini review. Front. Bioeng. Biotechnol..

[22] Fatima W., Batool S.R., Mushtaq F., Aslam M., Raza Z.A., Nazeer M.A. (2024). Controlled release of doxorubicin from gelatin-based nanoparticles: Theoretical and experimental approach. Mater. Adv..

[23] Vaghasiya K., Ray E., Singh R., Jadhav K., Sharma A., Khan R., Prakash O., Kumar R. (2021). Efficient, enzyme responsive and tumor receptor targeting gelatin nanoparticles decorated with concanavalin-A for site-specific and controlled drug delivery for cancer therapy. Mater. Sci. Eng. C.

[24] Zhou K., Zhu Y., Chen X., Li L., Xu W. (2020). Redox- and MMP-2-sensitive drug delivery nanoparticles based on gelatin and albumin for tumor targeted delivery of paclitaxel. Mater. Sci. Eng. C.

[25] Ibrahim H.M., Taha G.M., El-Alfy E.A., El-Bisi M.K. (2022). Enhancing antibacterial action of gauze by adding gelatin nanoparticles loaded with spectinomycin and chloramphenicol. Cellulose.

[26] Andrée L., Oude Egberink R., Dodemont J., Hassani Besheli N., Yang F., Brock R., Leeuwenburgh S.C.G. (2022). Gelatin Nanoparticles for Complexation and Enhanced Cellular Delivery of mRNA. Nanomaterials.

[27] Gisone I., Boffito M., Persiani E., Pappalardo R., Ceccherini E., Alliaud A., Cabiati M., Laurano R., Guiducci L., Caselli C. (2025). Integration of co-culture conditions and 3D gelatin methacryloyl hydrogels to improve human-induced pluripotent stem cells-derived cardiomyocytes maturation. Front. Bioeng. Biotechnol..

[28] Coester C.J., Langer K., Von Briesen H., Kreuter J. (2000). Gelatin nanoparticles by two step desolvation—A new preparation method, surface modifications and cell uptake. J. Microencapsul..

[29] Laurano R., Boffito M. (2020). Thermosensitive Micellar Hydrogels as Vehicles to Deliver Drugs With Different Wettability. Front. Bioeng. Biotechnol..

[30] (2009). Biological Evaluation of Medical Devices—Part 5: Tests for In Vitro Cytotoxicity.

[31] Laurano R., Torchio A., Ciardelli G., Boffito M. (2023). In Situ Forming Bioartificial Hydrogels with ROS Scavenging Capability Induced by Gallic Acid Release with Potential in Chronic Skin Wound Treatment. Gels.

[32] Boffito M., Gioffredi E., Chiono V., Calzone S., Ranzato E., Martinotti S., Ciardelli G. (2016). Novel polyurethane-based thermosensitive hydrogels as drug release and tissue engineering platforms: Design and in vitro characterization. Polym. Int..

[33] Zu G., Meijer M., Mergel O., Zhang H., van Rijn P. (2021). 3D-Printable Hierarchical Nanogel-Gelma Composite Hydrogel System. Polymers.

[34] Zatorski J.M., Montalbine A.N., Ortiz-Cárdenas J.E., Pompano R.R. (2020). Quantification of fractional and absolute functionalization of gelatin hydrogels by optimized ninhydrin assay and 1H NMR. Anal. Bioanal. Chem..

[35] Jahanban-Esfahlan A., Dastmalchi S., Davaran S. (2016). A simple improved desolvation method for the rapid preparation of albumin nanoparticles. Int. J. Biol. Macromol..

[36] Hickey R., Palmer A.F. (2020). Synthesis of Hemoglobin-Based Oxygen Carrier Nanoparticles by Desolvation Precipitation. Langmuir.

[37] Taguchi K., Chuang V.T.G., Hashimoto M., Nakayama M., Sakuragi M., Enoki Y., Nishi K., Matsumoto K., Seo H., Otagiri M. (2020). Characterization of bovine lactoferrin nanoparticle prepared by desolvation technique. Chem. Pharm. Bull..

[38] Khramtsov P., Burdina O., Lazarev S., Novokshonova A., Bochkova M., Timganova V., Kiselkov D., Minin A., Zamorina S., Rayev M. (2021). Modified desolvation method enables simple one-step synthesis of gelatin nanoparticles from different gelatin types with any bloom values. Pharmaceutics.

[39] Mahor A., Prajapati S.K., Verma A., Gupta R., Singh T.R.R., Kesharwani P. (2016). Development, in-vitro and in-vivo characterization of gelatin nanoparticles for delivery of an anti-inflammatory drug. J. Drug Deliv. Sci. Technol..

[40] Schrade S., Ritschl L., Süss R., Schilling P., Seidenstuecker M. (2022). Gelatin Nanoparticles for Targeted Dual Drug Release out of Alginate-di-Aldehyde-Gelatin Gels. Gels.

[41] Azimi B., Nourpanah P., Rabiee M., Arbab S. (2013). Producing gelatin nanoparticles as delivery system for bovine serum albumin. Iran. Biomed. J..

[42] Park H.E., Gasek N., Hwang J., Weiss D.J., Lee P.C. (2020). Effect of temperature on gelation and cross-linking of gelatin methacryloyl for biomedical applications. Phys. Fluids.

[43] Vinjamuri B.P., Papachrisanthou K., Haware R.V., Chougule M.B. (2021). Gelatin solution pH and incubation time influences the size of the nanoparticles engineered by desolvation. J. Drug Deliv. Sci. Technol..

[44] Alexa R.L., Iovu H., Ghitman J., Serafim A., Stavarache C., Marin M.M., Ianchis R. (2021). 3D-printed gelatin methacryloyl-based scaffolds with potential application in tissue engineering. Polymers.

[45] Ahsan S.M., Rao C.M. (2017). The role of surface charge in the desolvation process of gelatin: Implications in nanoparticle synthesis and modulation of drug release. Int. J. Nanomed..

[46] Pfeiffer C., Rehbock C., Hühn D., Carrillo-Carrion C., De Aberasturi D.J., Merk V., Barcikowski S., Parak W.J. (2014). Interaction of colloidal nanoparticles with their local environment: The (ionic) nanoenvironment around nanoparticles is different from bulk and determines the physico-chemical properties of the nanoparticles. J. R. Soc. Interface.

[47] Hassani Besheli N., Martens M., Macías-Sánchez E., Olijve J., Yang F., Sommerdijk N., Leeuwenburgh S.C.G. (2023). Unraveling the Formation of Gelatin Nanospheres by Means of Desolvation. Nano Lett..

[48] Ghosh R.N., Thomas J., Vaidehi B.R., Devi N.G., Janardanan A., Namboothiri P.K., Peter M. (2023). An insight into synthesis, properties and applications of gelatin methacryloyl hydrogel for 3D bioprinting. Mater. Adv..

[49] Vigata M., Meinert C., Bock N., Dargaville B.L., Hutmacher D.W. (2021). Deciphering the Molecular Mechanism of Water Interaction with Gelatin Methacryloyl Hydrogels: Role of Ionic Strength, pH, Drug Loading and Hydrogel Network Characteristics. Biomedicines.

[50] Yan X., Jia Y., Man H., Liu L., Sun S., Qi B., Li Y. (2023). Intermolecular interactions and gel properties of composite agglomerative networks based on oppositely charged polymers: Effects of pH and ionic strength. Food Hydrocoll..

[51] Ball V. (2024). Specific Ion Effects in Hydrogels. Molecules.

[52] Bose S., Phan C.-M., Rizwan M., Tse J.W., Yim E., Jones L. (2024). Fabrication and Characterization of an Enzyme-Triggered, Therapeutic-Releasing Hydrogel Bandage Contact Lens Material. Pharmaceutics.

[53] Zhu M., Wang Y., Ferracci G., Zheng J., Cho N.-J., Lee B.H. (2019). Gelatin methacryloyl and its hydrogels with an exceptional degree of controllability and batch-to-batch consistency. Sci. Rep..

[54] Kang M.G., Lee M.Y., Cha J.M., Lee J.K., Lee S.C., Kim J., Hwang Y., Bae H. (2019). Nanogels Derived from Fish Gelatin: Application to Drug Delivery System. Mar. Drugs.

[55] Wang J., Wang C., Wang Q., Zhang Z., Wang H., Wang S., Chi Z., Shang L., Wang W., Shu Y. (2022). Microfluidic Preparation of Gelatin Methacryloyl Microgels as Local Drug Delivery Vehicles for Hearing Loss Therapy. ACS Appl. Mater. Interfaces.

[56] Iqbal N., Bano A., Ahmed T., Nabeel M.I., Ullah J., Musharraf S.G., Saleem I., Malik M.I. (2025). Fish gelatin nanoparticles: An effective carrier for curcumin delivery. J. Drug Deliv. Sci. Technol..

[57] Kovács A.N., Katona G., Juhász Á., Balogh G.T., Csapó E. (2022). Albumin-hyaluronic acid colloidal nanocarriers: Effect of human and bovine serum albumin for intestinal ibuprofen release enhancement. J. Mol. Liq..

