# Supplementary material for: Design and Characterization of GelMA Nanogels (nanoGelMA) via Desolvation and Photopolymerization for Drug Delivery Applications
Source: Pharmaceutics. 2026 Jun 30;18(7):812. doi: 10.3390/pharmaceutics18070812 (PMC13416480; doi:10.3390/pharmaceutics18070812)
Supplement: Supplementary file 1 [file pharmaceutics-18-00812-s001.zip › pharmaceutics-4368160-supplementary.pdf]

Design and characterization of GelMA nanogels (nanoGelMA) via desolvation and photopolymerization for drug delivery applications

Table S1. Comparison of gelatin- and gelatin methacryloyl (GelMA)-based nanoparticle systems reported in the literature with respect to constituent material, preparation method, crosslinking strategy, and particle size.

Material	Preparation method	Crosslinking	Size	References
Gelatin	Two-step desolvation	Glutaraldehyde	~270 nm	Carvalho et al. [10]
Gelatin	One step desolvation	Glutaraldehyde or genipin	~250 nm	Park et al. [12]
Gelatin	Emulsification	Glutaraldehyde	100-200 nm	Cascone et al. [13]
GelMA	Emulsification	Photo-crosslinking	~200 nm	Kim et et al. [14]
Gelatin	Emulsification	Genipin	100 nm	Choubey & Bajpai [15]
Gelatin	Two-step desolvation *vs.* Nanoprecipitation	Glutaraldehyde	100-200 nm	Khan & Schneider [16]
Gelatin	Nanoprecipitation	Glutaraldehyde	~200 nm	Lee et al. [17]
Gelatin	Two-step desolvation	Glutaraldehyde	200-400 nm	Fatima et al. [22]
Gelatin	Two-step desolvation	Glutaraldehyde	~200 nm	Vaghasiya et al. [23]
Gelatin	One-step desolvation	Glutaraldehyde	~250 nm	Ibrahim et al. [25]
Gelatin	Two-step desolvation	Glutaraldehyde	~400 nm	Andrée et al. [26]
Gelatin	Two-step desolvation	Glutaraldehyde	200-300 nm	Coester et al. [28]
Gelatin	One-step desolvation	Glutaraldehyde	100-400 nm	Khramtsov et al. [38]
GelMA	Emulsification	Photo-crosslinking	~200 nm	Kang et al. [54]
GelMA	Two-step desolvation	Photo-crosslinking	~250 nm	**Present work**

References listed in the manuscript.

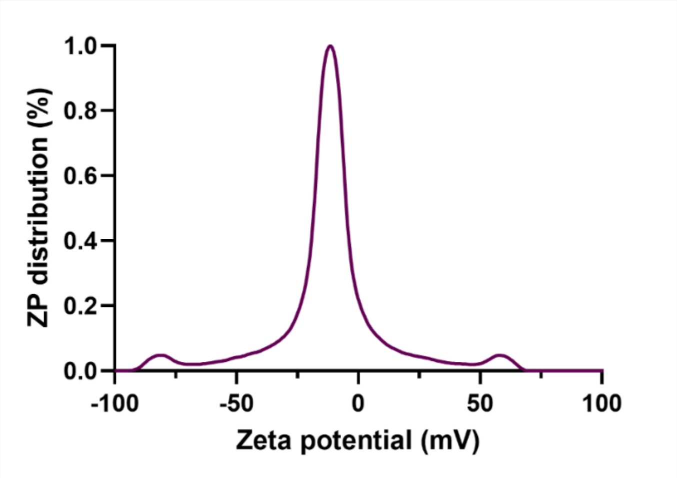

Figure S1. Average Zeta potential distribution measured on a representative batch of nanoGelMA dispersion in ddH_2O.
